# Feasibility and safety of high-intensity interval training for the rehabilitation of geriatric inpatients (HIITERGY) a pilot randomized study

**DOI:** 10.1186/s12877-020-01596-7

**Published:** 2020-06-05

**Authors:** Rita Pires Peixoto, Véronique Trombert, Antoine Poncet, Jérôme Kizlik, Gabriel Gold, Georg Ehret, Andrea Trombetti, Jean-Luc Reny

**Affiliations:** 1 Division of Internal Medicine for the elderly, Trois-Chêne, Department of Rehabilitation and Geriatrics, Geneva University Hospitals and Faculty of Medicine, Geneva, Switzerland; 2 Division of Cardiology, Department of Medicine, Geneva University Hospitals and Faculty of Medicine, Geneva, Switzerland; 3 Division of clinical epidemiology, Geneva University Hospitals and Geneva Faculty of Medicine, Geneva, Switzerland; 4 Division of Bone Diseases, Department of Medicine, Geneva University Hospitals and Faculty of Medicine, Geneva, Switzerland; 5 Division of General Internal Medicine, Department of Medicine, Geneva University Hospitals and Faculty of Medicine, Rue Gabrielle Perret Gentil 4, 1211 Geneva, Switzerland

**Keywords:** HIIT, High-intensity interval training, Rehabilitation, Elderly, Feasibility, Safety

## Abstract

**Background:**

High-intensity interval training (HIIT) has been shown to be more effective than moderate-intensity continuous training (MICT) for the physical rehabilitation. However, data on its suitability for older hospitalized patients is scarce.

**Methods:**

Randomized controlled trial in a hospital setting. Inclusion of 100 patients, ≥65 years old, hospitalized for rehabilitation after an acute medical condition, in a two-week rehabilitation program of either four HIIT or three MICT sessions per week. Completion was defined as participation in all but two planned sessions accomplishing ≥50% of each session. We assessed: upper-limb muscle strength (handgrip isometric strength test), lower-limb muscle strength (quadriceps and ankle flexion and extension tests); gait speed and spatio-temporal parameters (instrumented walkway), and exercise capacity (6-min walk test). All adverse events were recorded as safety endpoints.

**Results:**

An intention-to-treat analysis showed a 44% completion rate for the HIIT group (95% CI, 30–59) and 77% for MICT (95% CI, 55–82). A modified intention-to-treat analysis restricted to patients who participated in ≥1 session showed an 88% completion rate in the HIIT group (95%CI, 69–97) and an 80% completion rate in MICT (95%CI, 65–90). The exercises most frequently undertaken were the pedal exerciser (54%) and the NuStep (32%). There were no significant differences in the various measures. No serious adverse events occurred.

**Conclusion:**

A HIIT rehabilitation program for this population was feasible, safe and had a high adherence rate.

**Trial registration number:**

Clinicatrials.gov ID: NCT02318459.

**Trial registration date:** November 7th, 2014. Retrospectively registered.

This study adheres to the CONSORT guidelines.

## Background

Aerobic exercise provides several physical and psychological benefits important to geriatric patients, such as mobility, cognition, mood, and cardiovascular disease [1]. However, the most effective approach for this population remains uncertain. Recent evidence suggests that high-intensity, high-dose, strength, aerobic, and balance training can improve both functional capacity and quality of life among older adults [2]. With growing numbers of older patients in hospitals, rehabilitation allowing prompt, safe discharge home has become increasingly important for this often frail population. Most available studies on geriatric rehabilitation have focused on reducing the risk of falls, but also showed that supervised, group exercise programs were effective at improving physical performance [3] and functional capacity [4], and may be more cost-effective [5].

High-intensity interval training (HIIT) is a method that splits exercise sessions into several intervals, alternating high-intensity exercise for several seconds or minutes with active or passive rest. This allows the cardiovascular system incomplete—but sufficient—recovery before a new high-intensity interval. Sessions last from 8 to 25 min of actual exercise [5–8]. This approach stimulates the peripheral muscles without inducing excessive cardiac stress [9], helps participants reach a higher percentage of peakVO_2_ [7] and exercise capacity [10], and achieves the same work volume more quickly than moderate-intensity continuous training (MICT).

Interestingly, the mean age in HIIT trials rarely exceeds 70 years old, with few specific studies targeting older populations [5, 11]. Whether HIIT is a feasible, safe, and beneficial approach for elderly patients hospitalized after an acute medical event is unknown. We assessed the feasibility of a HIIT-based rehabilitation program in comparison to a conventional MICT approach.

## Methods

### Participants

The HIITERGY study is a pilot randomized study designed to assess the feasibility of a HIIT program and compare it to a conventional MICT rehabilitation program for patients ≥65 years old, hospitalized for rehabilitation after an acute medical condition. Consecutive patients were invited to participate if they were: expecting rehabilitation lasting ≥2 weeks; able to follow instructions for a Timed Up and Go (TUG) test [12] and perform the proposed exercises; and willing to participate in 4 HIIT sessions per week for 2 weeks.

Early study termination criteria were any acute or unstable medical or surgical condition, an abnormal exercise stress test (EST), delirium, inability to follow instructions, or inability to give consent. Full inclusion and exclusion criteria are listed in Additional Table 1.

**Table 1:** Exercises used in the HIIT group

	Exercise A	Exercise B	Exercise C	Exercise D
**Upper-body options**	- NuStep	- Two-armed abduction over head - Two-armed abduction to shoulder level - Two-armed abduction to maximum level	- Two-armed extension over head - Two-armed extension to shoulder level - Two-armed extension to maximum level	- Alternating torso twist to same side as step - Alternating both hands to opposite hip
**Lower-body options**	- cycle ergometer - pedal exerciser	- Step up + step down - Step front + step back	- Squat - Sit on chair + get up (with or without help from arms)	- Side-step right + side-step left - Foot touch floor right + foot touch floor left

From May 2014 to November 2015, we enrolled 100 patients from Geneva University Hospitals’ Division of Internal Medicine and Rehabilitation and Division of Geriatrics. Subjects were randomized using a computerized random-number generator, to the HIIT or MICT programs, in block sizes of 4 with allocation concealment. Sealed envelopes were prepared by an assistant independent of the study. RPP and VT enrolled the participants and assigned them their allocated groups after randomization. The study ended after the pre-specified number of patients were included. All clinical events during the exercise sessions were recorded in the clinical report form by investigators. All clinical events after the session were monitored through the electronic medical record, including systematic report on falls, for example.

The study was performed according to the principles of the Declaration of Helsinki and was approved by the Geneva Medical Research Ethics Committee (CEREH *n*°13–257). Written informed consent was obtained from all patients. Consent to publish was obtained from the patient seen in videos 1 and 2.

### Exercise stress testing

EST was only performed on patients randomized to the HIIT group. EST was individualized for each patient and measured maximum heart rate (maxHR), excluded those with positive tests, and evaluated each one’s capacity for exercise [13, 14]. The rate of perceived exertion (RPE), assessed using the original Borg scale, guided the test’s progression (see also text).

### Exercise training

Detailed information regarding exercise training programs is available online. HIIT exercises are described in Table 1.

Patients randomized to HIIT had four 30-min, group sessions per week (Monday, Tuesday, Thursday, and Friday) for 2 weeks, with a maximum of 4 patients per group. Patients were supervised using heart-rate monitors (POLAR RS800CX, Polar Electro Europe AG, Zug, Switzerland) to help them reach but not exceed about 95% of the maxHR, with an RPE target of 8–9/10 on the modified Borg scale [13, 15] (Additional Figs. 1 and 2). HIIT sessions took place under the supervision of a physical therapist and a physician, for quality control and safety.

Patients randomized to MICT had three 40-min, group sessions per week (Monday, Wednesday, and Friday) for 2 weeks, with a maximum of 10 patients per group. Patients were supervised using heart-rate monitors (Additional Figs. 3 and 4). MICT sessions took place under the supervision of a physical therapist.

### Main outcome

Feasibility criteria were pre-defined as completion of all but two planned sessions, plus completion of ≥50% of each session.

### Follow-up and secondary clinical endpoints

#### Functional measurements

All patients were clinically evaluated at the beginning and end of the study. This involved: resting ECG and blood pressure measurements; weight and height; a walking capacity exercise using the 6-min walk test (6MWT); muscle strength tests using the JAMAR® portable dynamometer (Sammons Preston Rolyan, Bolingbrook, IL, USA) for handgrip isometric strength and the MicroFET2™ portable dynamometer (Biometrics, Gometz-le-Châtel, France) for quadriceps flexion and extension plus ankle flexion and extension; and a gait analyis of spatio-temporal gait parameters (inlcuding step time and length variability) using the GAITRite® instrumented walkway (Biometrics, Gometz-le-Châtel, France). Due to difficulties to assess strength with the JAMAR® dynamometer for the first 19 patients (readings under 1 kgf on a scale of 1 to 90) we switched to the Vigorimeter® dynamometer (KLS MARTIN Group, Mulhouse, France) for the next 81 patients. Maximum muscle strength between both sides was recorded, which in turn was the mean value among three measurements.

#### Safety endpoints

Pre-defined safety endpoints included the monitoring of falls during the study sessions; drop-out due to pain, fear, or fatigue; musculoskeletal injury; any cardiac event (acute coronary syndrome, arrhythmia, cardio-respiratory arrest, sudden death); and respiratory symptoms (dyspnea, cough, bronchospasm).

#### Criteria for a patient to stop the study

Any patient presenting an exclusion criterion, a safety endpoint, refusing the initial assessment, not attending more than 2 exercise sessions, or wishing to stop the study was immediately evaluated by a physician before leaving the study. Data from these patients were censored at the date of the last session and included in the modified intention-to-treat analysis (mITT) provided that at least one session of HIIT or MICT had been completed.

### Intention to treat and modified ITT populations

ITT population consisted of all patients enrolled and randomized in the study, including patients who did not start the program, for whatever reason.

mITT population was restricted to the patients who actually initiated their program and performed at least one session.

### Statistical analysis

Although based on a randomized design the present study aims primarily at assessing the feasibility and safety of HIIT, not to prove a difference compared to MICT. The MICT group was included to provide reliable descriptive and hypothesis generating data for future randomized controlled trials (RCT). Thus the sample size was estimated for a target precision of 10% for the proportion of patients who could complete the HIIT program (half-width of the 95% CI). Between 25 and 96 patients would thus be needed to allow us to observe a completion rate ranging for HIIT from 93% (80–100) to 50% (40–60), respectively. Based on these estimations, we aimed to include 50 patients in each group.

Patients’ characteristics and outcomes are described as frequencies and percentages for qualitative variables and as means and standard deviations for quantitative variables.

The feasibility analysis was performed on ITT and mITT populations. The Clopper-Pearson exact method was used to estimate 95% CI of the feasibility proportion in each group.

For secondary clinical outcomes (6MWT, spatio-temporal gait parameters, muscle strength), the overall training effect (i.e., the performance difference between the first and last session) within group was assessed using a linear mixed-effects regression model, with patients as the random effect and the session factor as the fixed effect. The equation model can be written as Performance = β_0_ + β_1_ Session + ε, where the coefficient of interest, β_1_, represents the overall training effect. These analyses were performed on the ITT population only. Of note, only patients assessed with Vigorimeter® dynamometer were included in the analysis of maximal muscle strength.

With respect to the study objective and the potential selection bias related to the EST only being performed on the HIIT group, statistical comparisons between HIIT and MICT groups were not relevant.

Data were recorded using a secuTrial® database (version 4.7.1.7, 2014, Berlin, Germany) and were analyzed using R software, version 3.3.1 (R Foundation for Statistical Computing, Vienna, Austria). All analyses were assessed at a two-sided alpha level of 5%.

## Results

### Patients

**Fig. 1:**
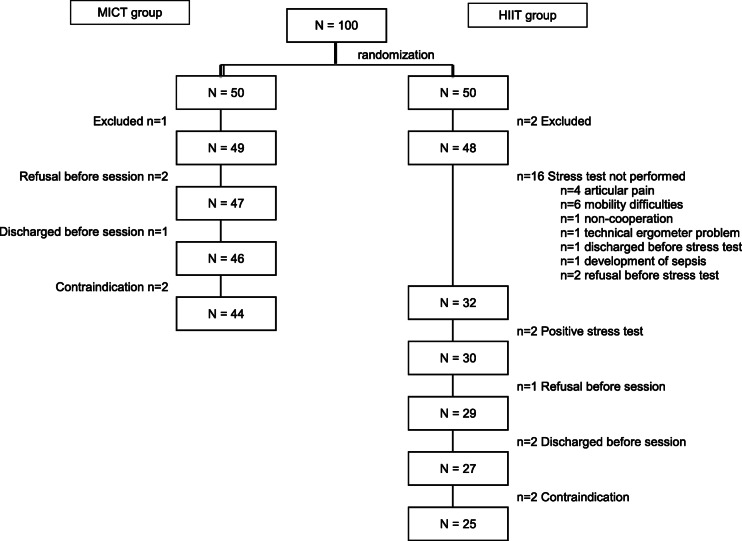
Study flow chart

A study flow-chart is shown in Fig. 1. Two HIIT patients were excluded due to criteria missed at inclusion but detected before the EST, 16 were unable to perform the EST, and two failed the test—one with ST-segment modifications and another with a blood pressure drop during exercise. Neither suffered a cardiovascular event during follow-up. These 20 patients were not allowed to participate in HIIT sessions and were invited to follow conventional rehabilitation programs outside the trial. After the EST and before the first session, one patient refused participation, two were discharged from hospital, and two were excluded due to the occurrence of an exclusion criterion. Thus, 25 patients began the program and participated in at least one HIIT session; their demographic characteristics were similar to those of the 25 excluded patients.

From the initial 50 MICT group patients, six were excluded, leaving 44 who began the program and participated in at least one MICT session.

Patients’ characteristics of the ITT population are outlined in Table 2.

**Table 2:** Patients’ characteristics and types of HIIT exercises

	All pts (***n*** = 100)	HIIT (***n*** = 50)	MICT (***n*** = 50)
Women, *n* (%)	65 (65%)	34 (68%)	31 (62%)
Age (y), mean ± SD	85 ± 7	85 ± 7	84 ± 7
Age (y), min - max	67–102	67–102	70–101
BMI (kg/m2), mean ± SD	25 ± 6	25 ± 5	25 ± 6
Heart rate (/min), mean ± SD	73 ± 11	73 ± 10	73 ± 11
Systolic BP (mmHg), mean ± SD	135 ± 16	138 ± 14	133 ± 17
Diastolic BP (mmHg), mean ± SD	68 ± 13	69 ± 14	68 ± 12
Main problem at inclusion visit, *n* (%)			
Cardiopulmonary problem	11 (11%)	6 (12%)	5 (10%)
Deconditioning	14 (14%)	7 (14%)	7 (14%)
Gait impairment/falls	53 (53%)	27 (54%)	26 (52%)
Neurological problem	6 (6%)	4 (8%)	2 (4%)
Other	16 (16%)	6 (12%)	10 (20%)
Sessions			
Scheduled	464	200	264
Not participated in	70	25 (13%)	45 (17%)
Early hospital discharge	36	17	19
Early hospital transfer	10	0	10
Competing consultation/exam	9	3	6
Home visit	2	2	0
Session cancelled by staff	2	2	0
Acute gastroenteritis	1	1	0
Exclusion due to complications or contra-indications	5	0	5
Patient transport issues	2	0	2
Family meeting	2	0	2
Fall	1	0	1
Participated in	394	175 (88%)	219 (83%)
Completed	320	157 (90%)	163 (74%)
Not completed	74	18 (10%)	56 (26%)
Patient refusal	49	16	33
Patient unavailability	4	2	2
Knee pain	6	0	6
Incapacity to understand instructions	5	0	5
< 50% of session completed	7	0	7
Exclusion due to suspected sacral fracture	2	0	2
Dizziness and malaise	1	0	1
Type of HIIT exercise (157 completed sessions)			
A		147 (94%)	
pedal exerciser		79 (54%)	
NuStep		47 (32%)	
cycle ergometer		21 (14%)	
B		5 (3%)	
C		4 (3%)	
D		1 (1%)	

*HIIT* High-intensity interval training, *MICT* Moderate-intensity continuous training, *SD* standard deviation

### Clinical outcomes

#### Main outcome

Among the exercises available for HIIT patients (Table 1), Type A exercises, based on the pedal exerciser, NuStep, or cycle ergometer, were the most frequent of all completed sessions (Table 2). A typical HIIT session is available in Additional Video 1.

Based on the study definition, 22 of the 25 patients who started the HIIT program successfully completed it. Out of 200 planned HIIT session participations, 175 were scheduled (87.5%), of which 157 (90%) were completed (Table 2).

Based on the study definition, 35 of the 44 patients who started the MICT program successfully completed it. Out of 264 planned MICT session participations, 219 were scheduled (83%) and 163 (74%) were completed (Table 2).

In the ITT analysis, 44% (95% CI, 30–59) of HIIT patients and 70% (95% CI, 55–82) of MICT patients were deemed to have successfully completed their respective rehabilitation programs. Thus, overall, 39% (157/400) of scheduled HIIT session participations were completed, and 54% (163/300) of scheduled MICT session participations were completed.

Using the mITT analysis, however, 88% (95% CI, 69–97) of HIIT patients and 80% (95% CI, 65–90) of MICT patients were deemed to have successfully completed their respective rehabilitation programs.

#### Secondary clinical outcomes

Patients in the mITT population were monitored for secondary clinical outcomes: 6MWT, spatio-temporal gait parameters and muscle strength. Table 3 presents their performances at the first and last sessions. As the aim of the study was not to show the superiority of one rehabilitation mode over another, data of the two groups was pooled to describe the overall evolution of various functional, gait and strength variables. Of note there was no meaningful difference between HIIT and MICT.

**Table 3:** 6-min walk test, gait parameters and maximal muscle strength

mITT population (***n*** = 69)	First visit	Last visit	Mean difference^**$**^ (95% CI)	***P***value*
**6-min walk test**	**(*N*** **= 51)**	**(*N*** **= 38)**	**(*N*** **= 54)**	
Distance	213 ± 93	257 ± 79	23 (6 to 39)	0.008
Predicted (%)	59 ± 39	76 ± 38	8 (2 to 14)	0.007
**Spatio-temporal gait parameters**				
**Usual pace**	**(*N*** **= 56)**	**(N = 38)**	**(*N*** **= 62)**	
Speed (cm/sec)	56 ± 22	62 ± 20	4.6 (−0.8 to 10.0)	0.093
Mean step length (cm)	37 ± 10	39 ± 10	2.2 (0.0 to 4.4)	0.048
Mean stride length (cm)	74 ± 21	77 ± 20	3.6 (−1.3 to 8.5)	0.142
Mean support base (cm)	13 ± 4	14 ± 4	− 0.1 (− 1.1 to 0.9)	0.801
Mean double support time (msec)	5.8 ± 2.3	5.1 ± 1.5	− 0.5 (− 0.9 to 0.0)	0.053
**Maximum pace**	**(*N*** **= 57)**	**(*N*** **= 39)**	**(*N*** **= 61)**	
Speed (cm/sec)	77 ± 31	85 ± 29	8.5 (3.6 to 13.4)	0.001
Mean step length (cm)	42 ± 13	45 ± 13	2.9 (0.7 to 5.1)	0.011
Mean stride length (cm)	85 ± 26	91 ± 26	6.0 (1.6 to 10.3)	0.008
Mean support base (cm)	13 ± 4	13 ± 4	−0.7 (−1.6 to 0.2)	0.105
Mean double support time (msec)	4.3 ± 2.1	3.8 ± 1.4	−0.3 (− 0.6 to − 0.1)	0.013
**Gait variability parameters**				
**Usual pace**	**(*N*** **= 55)**	**(N = 38)**	**(N = 62)**	
CV step length	13.2 ± 6.8	12.2 ± 6.1	−0.7 (−3.0 to 1.5)	0.503
CV step time	8.8 ± 7.4	7.6 ± 2.9	−0.4 (−1.7 to 0.8)	0.502
CV stride length	7.7 ± 4.0	7.5 ± 3.8	− 0.1 (− 1.7 to 1.4)	0.846
CV stride time	5.7 ± 5.4	4.7 ± 2.0	−0.4 (− 1.3 to 0.5)	0.361
**Maximum pace**	**(N = 57)**	**(N = 39)**	**(N = 61)**	
CV step length	11.7 ± 7.5	11.6 ± 10.1	0.2 (−3.2 to 3.5)	0.914
CV step time	8.5 ± 8.6	6.7 ± 2.9	−0.3 (−1.2 to 0.7)	0.590
CV stride length	6.7 ± 3.2	7.1 ± 5.5	0.4 (−1.2 to 2.0)	0.613
CV stride time	4.7 ± 3.7	5.0 ± 4.9	0.5 (−1.0 to 2.0)	0.504
**Maximal muscle strength (kPa)**	**(*N*** **= 46)**	**(*N*** **= 35)**	**(N = 50)**	
Handgrip	33.3 ± 15	29.7 ± 18.7	−0.2 (−2.9 to 2.6)	0.895
Knee flexion	12.5 ± 3.6	12.9 ± 4.2	0.4 (−1.0 to 1.8)	0.564
Knee extension	14.8 ± 4.8	15.8 ± 5.0	0.8 (−1.1 to 2.8)	0.392
Ankle dorsiflexion	19.2 ± 6.7	20.7 ± 7.4	1.4 (−1.1 to 3.8)	0.254
Ankle plantar flexion	18.7 ± 8.0	18.2 ± 6.6	−0.7 (−3.3 to 1.8)	0.553

^$^Mean difference (last visit – first visit) estimates and pvalues from linear mixed-effects models

*CV* Coefficient of variation (dimensionless)

Maximal muscle strength among both sides (kPa) assessed with a Vigorimeter® dynamometer

Overall, despite remaining below 300 m, distance walked significantly increased, for the overall study population (pooled MICT and HIIT data), by an average of 23 m between visits, corresponding to an 8% average increase in predicted walking distance. Gait parameters significantly improved at maximum pace, except mean support base. Similar results were observed at usual pace, but differences failed to reach statistical significance. Gait variability data, at usual pace as at maximum pace, did not show any statistical difference between the first and last session. We observed no differences in maximum muscular strength between first and last visits.

Using multivariable mixed effects model including an interaction term between group factor and session factor, none of those outcomes showed evidence of a different trend evolution (between first and last session) between HIIT and MICT groups.

##### Adverse events

There were no serious adverse events in either group during study sessions (Table 4). In the mITT analysis, the proportions of patients showing complications in the HIIT and MICT groups were 64% (16/25) and 36% (16/44), respectively, mostly driven by osteoarticular pain in HIIT patients. Only one HIIT patient (4%) stopped a session due to a minor complication while 7 MICT patients (16%) stopped several sessions due to minor complications.

**Table 4:** Complications during sessions among patients completing ≥1 session

	All patients (*n* = 69)	HIIT (*n* = 25)	MICT (*n* = 44)
Any complication	32 (46%)	16 (64%)	16 (36%)
Osteoarticular pain	14 (20%)	10 (40%)	4 (9%)
Fatigue	5 (7%)	2 (8%)	3 (7%)
Dyspnea	4 (6%)	2 (8%)	2 (5%)
Dizziness/malaise	4 (6%)	3 (12%)	1 (2%)
Muscular pain	4 (6%)	3 (12%)	1 (2%)
Fall	2 (3%)	1 (4%)	1 (2%)
Musculoskeletal lesion	1 (1%)	1 (4%)	0
Thoracic pain	1 (1%)	0	1 (2%)
Other complication	7 (10%)	2 (8%)	5 (11%)
Refusal of session	2 (3%)	0	2 (5%)
Session stopped due to complication	8 (12%)	1 (4%)	7 (16%)

*HIIT* High-intensity interval training, *MICT* Moderate-intensity continuous training

## Discussion

The present study showed that HIIT was safe and feasible in a supervised setting for a group of carefully screened patients aged 67–102 years old shortly after an acute medical event. Of those able to begin a session, adherence to the whole program was 88% for HIIT versus 80% for MICT. Importantly 18/50 (36%) of included patients could not participate in the HIIT group, either because the EST was not considered normal (2/50 or 4%) or because patients were unable to complete the EST (16/50 or 32%).

On average, HIIT participants seemed to attend and complete proportionally more sessions than MICT participants. In other studies, younger subjects tended to enjoy HIIT [7, 8, 16], leading to a longer duration of physiological changes [17–19]. HIIT interventions shorter than 6 weeks were unlikely to show clinically relevant physiological changes [16].

The 1 min of effort for 1 min of passive rest-type protocol chosen proved to be feasible. It has been suggested previously that starting with short active- and short passive rest intervals is effective for less fit or cardiovascular patients [9, 20–22]. The actually intense nature of the effort in the HIIT group was closely monitored with the Borg scale and continuous heart rate recording. Rest periods of 24 h–72 h in the present study were sufficient for recovery, without patients showing signs of reduced work capacity, fatigue, or overtraining; a previous study had suggested minimum rest periods of 5 days [23].

The cycle ergometer has been described as a more favorable mode of aerobic exercise among geriatric patients, due to the reproducibility and easier monitoring of effort intensity results compared to callisthenics, which are more common in chronic heart failure rehabilitation programs [24]. We noted that exercises performed using stationary machines (pedal exerciser, NuStep, cycle ergometer) were suitable and safe for this population at risk of falls. The NuStep has the additional advantage of being a full-body trainer [25]. The other exercises proved to be more difficult due to balance, gait, and coordination issues, and they did not increase HR into the desired range. This selection of relevant exercices could be useful for the design of HIIT programs for geriatric populations.

It has been demonstrated that HIIT improves subjects’ functional capacity, increasing distances on the 6MWT [5, 21], muscle strength [26], mechanical efficiency during walking [27], and other parameters [5, 27]. In our study, the mean distance on the first 6MWT was below 300 m, pointing to a frail population [28] despite remaining non-specific and non-diagnostic [29]. Mean distance increased by an average of only 23 m—below the threshold for substantial clinical significance [30]. The increase in predicted walked distance, which is better correlated to functional status [31], also increased significantly (mean, 8%).

Certain gait parameters of older patients are significant predictors of fall risk [32], and gait speed seems to be a reliable and sensitive measure [33]. The average usual pace throughout the present study was lower than expected in a healthy elderly population and in those transitioning to frailty [34], which may be explained by their recent acute medical event. Only the gait parameters at maximum speed showed a significant improvement at the end of the program, but not the gait variability parameters. A meta-analysis showed that HIIT did not improve gait speed any more than MICT [35].

Our intention was not to prove differences between these programs but to evaluate the safety and feasibility of HIIT and of muscle strength assessment in the oldest old. Handgrip strength was initially measured using the JAMAR® dynamometer, which had to be switched to the Vigorimeter®, as mentioned, in order to provide adequate measurement; our baseline handgrip strength results were comparable to a previous publication using the Vigorimeter® [36]. These dynamometers have correlated well in different populations [36], but in older patients, the JAMAR® device may present handling difficulties due to its rigidity and weight [37], as was best evidenced in this study population.

HIIT has a few limitations and constraints in terms of means of exercise: it requires a good level of motivation and program adherence, a potential need for medical approval including an EST, and initial supervision for non-trained individuals [38]. High-impact exercises may not suit everybody, but protocols can be adapted to derive the same benefits. HIIT sessions provided a good subjective level of satisfaction, though not quantified, even among the very oldest participants, as best shown in Additional Video 2 for a 102 year-old patient. This was in line with previous studies on adherence to HIIT programs versus lower-intensity methods in populations of much younger patients [17, 18].

It is of note that overall weekly session duration was the same for both groups (120 min/week), although active exercise time was shorter in the HIIT than in the MICT program (72 vs. 120 min/week). This may have had an impact on the adherence of unfit patients.

Supervised HIIT programs applied to different populations have been shown to be safe, with a low risk comparable to that of traditional exercises [7, 8, 39, 40] but specific data were lacking in the oldest old. Although sample size is small, this study showed that HIIT was safe in this population. Indeed, the HIIT group experienced expected minor osteoarticular pain but did not experience severe adverse events.

An increased risk of falls among older adults following participation in a HIIT session was previously reported [41]. The number of falls during sessions in both of our groups was low, with only one near-fall in the HIIT group at the end of a session while the patient was getting off the cycle ergometer.

### Future perspectives

While multicomponent exercise intervention including low-intensity resistance training recently proved to be safe and effective to reverse the functional decline associated with acute hospitalization in elderly patients [42], this line of research deserves further RCTs on the potential benefits of HIIT interventions for older patients who need a rehabilitation program following an acute hospitalization. The present feasibility study provides some insight into the design of such RCTs: i) the need to perform an EST on all patients before randomization to avoid selection bias; ii) a longer program duration (6–12 weeks) would allow assessment of impacts on strength and function using appropriate dynamometers; iii) ideally, an assessment of peakVO2 could evidence improvements in functional capacity; iv) and finally, an evaluation of impacts on frailty could also point to other advantages of this type of intervention in populations at risk.

### Limitations

The EST excluded 36% (18/50) of our potential HIIT participants. This may have induced a selection bias in favor of a fitter population in the HIIT group. The main objective of the EST was to determine the 95% maxHR not to be exceeded during HIIT sessions and, due to a questionable risk/benefit balance, we chose not to perform the EST on MICT patients. Our main goal was the feasibility of HIIT using commonly available tools and indicators such as the Borg scale as surrogates. Ideally, VO2peak and the VCO2/VO2 ratio should have been measured during the initial stress test to affirm that the effort was really maximal. However, VO2max test could possibly exclude even more patients due to a predicted higher rate of unaccomplished tests, and so limit the access of potential candidates. It could rather be included as an outcome in a trial assessing the superiority of HIIT compared to MICT. Similarly, in a future RCT, the EST should be offered to patients of both HIIT and MICT arms and primarily used as a measure of the maximal HR and corresponding Borg scale value. Based on the experience with this pilot study, we suggest that the inability of a patient to perform an EST should not become an exclusion criterion for HIIT. Only abnormal EST should trigger, if relevant, further investigations and preclude HIIT based on clinical exclusion criteria. This pragmatic approach is supported by the proven safety of HIIT in population at high risk of malignant arrhythmias [7, 8, 39, 40]. Ultimately, if an adequately powered RCT confirms the absence of malignant arrhythmias, the requirement for an EST should be abandoned for routine HIIT, provided HIIT becomes an option.

The resources used in the HIIT group (an EST, the presence of a physician during the exercise sessions) were greater than in the MICT group in this study, due to safety concerns. However, if an adequately powered RCT confirms the safety of HIIT, these additional resources would not be required and it could become an alternative to MICT at no additional expenses.

Two weeks of training may appear unusual but this was constrained by the mean length of stay of our patients and within the frame of a feasibility study rather than a superiority trial.

The number of patients unable to complete the study due to competing organizational issues, such as consultations and early discharges, was relatively high in both groups, but this reflects real life.

## Conclusion

HIIT can be applied to older inpatients who follow the program with high rates of adherence. It is both a feasible and safe strategy in a supervised hospital setting when appropriate exclusion criteria are applied.

## Supplementary information

**Additional file 1.**

**Additional file 2.** Video 1 - A typical HIIT session with a 102 year-old patient. Excerpt from a typical HIIT session using a pedal exerciser. NB: the intervals of exercise and rest and the use of the modified Borg scale to assess and adapt the exercise intensity according to the patient’s perceived level of exertion.

**Additional file 3.** Video 2 - An interview with the same 102 year-old patient. A spontaneous testimony of the patient’s appreciation of the nature and effects of the HIIT program, as well its potential benefits during geriatric hospitalization. This video sequence was recorded in a single shooting.

## Data Availability

The datasets used and/or analyzed during the current study are available from the corresponding author on reasonable request.

## Abbreviations

6MWT: 6-min walk test
EST: Exercise stress test
HIIT: High-intensity interval training
ITT: Intention to treat
kPa: Kilopascal
MICT: Moderate-intensity interval training
maxHR: Maximal heart rate
mITT: Modified intention to treat
peakVO2: Peak oxygen consumption
RPE: Rate of perceived exertion
TUG: Timed up and go
