# Supplementary material for: Feasibility and safety of high-intensity interval training for the rehabilitation of geriatric inpatients (HIITERGY) a pilot randomized study
Source: BMC Geriatr. 2020 Jun 5;20:197. doi: 10.1186/s12877-020-01596-7 (PMC7275527; doi:10.1186/s12877-020-01596-7)
Supplement: Supplementary file 1 — Additional file 1. [file 12877_2020_1596_MOESM1_ESM.docx]

**Additional material**

Additional text .................................................................................................................................................. 2

Exercise Stress Test (EST)

Exercise training

Statistical analysis

Additional Table 1. Inclusion and exclusion criteria .............................................................................................. 3

Additional Figure 1. HIIT session examp............................................................................................................... 4

Additional Figure 2. HIIT session example 2 ......................................................................................................... 5

Additional Figure 3. MICT session example 1 ....................................................................................................... 6

Additional Figure 4. MICT session example 2 ....................................................................................................... 7

**Additional text**

**Exercise Stress Test (EST)**

The EST was performed on either an upright or an inclined cycle ergometer, using a ramp test increasing by 25W resistance every 2 minutes (25-2-25R). Rate of perceived exertion (RPE) was used to guide the test because it is not influenced by dysrhythmia or beta-blockers (thus being more relevant than max HR) when determining maximum or sub-maximum exercise capacity.

**Exercise training**

**HIIT group**

The HIIT sessions consisted of a 10-minute warm-up (on a cycle ergometer, NuStep, or pedal exerciser), 15 minutes of HIIT (8 one-minute intervals of active exercise alternating with 7 one-minute intervals of passive rest), and 5 minutes of passive cool-down, making a total of 30 minutes per session (18 minutes of active exercise) and 120 minutes per week (72 minutes of active exercise).

At the initial evaluation, patients tested four different exercises (A–D) to evaluate their performance capacity, and the exercises were adapted to patients’ abilities and limitations (Table 2).

The patient’s RPE, measured according to a modified Borg scale, was recorded at baseline (before the beginning of the session), after the warm-up, after each effort interval, and after the cool-down period. The intensity of the exercise was adjusted after each interval.

**MICT group**

The MICT sessions consisted of a 10-minute warm-up (exercises targeting the upper and lower body), 25 minutes of Pedal Exerciser, and 5 minutes of active cool-down, making a total of 40 minutes per session (40 minutes of active exercise) and 120 minutes per week (120 minutes of active exercise). Exercises targeted muscular reinforcement, flexibility, balance, and aerobics, and intensity was adjusted using the Talk-Test, in which the patient must be able to maintain a conversation while exercising.

RPE was recorded, as per usual care, at baseline, during the pedal exerciser exercise, and after the cool-down.

**Statistical analysis**

Patients’ performances (6MWT, step variability, and muscle strength) at their first and last visits were described as means and standard deviations, based on available data. The overall training effect, i.e., the performance difference between first and last visits, was assessed using a linear mixed-effects regression model, with patients as the random effect and the visit factor as the fixed effect. The equation model can be written as Performance = β_0_ + β_1_ Visit + ε, where the coefficient of interest, β_1_, represents the mean change score (last visit – first visit).

The group effect on patients’ performances was evaluated as the difference in mean change score between the first and last visits. For this purpose, and for each performance outcome, we used a linear mixed-effects regression model where the parameter of interest was the interaction term between group factor and time (i.e., visit) factor. Group and time factors were the fixed effects and the patient was the random effect. The equation model can be written as Performance = β_0_ + β_1_ Group + β_2_ Visit + β_3_ Group*Visit + ε, where the coefficient of interest, β_3_, represents the difference between the mean change scores of each group. The training effect by group was estimated according to this model as β_2_ in HIIT group and β_2_ + β_3_ in MICT group.

**Additional figures and tables**

Table 1. Inclusion and exclusion criteria

| **Inclusion criteria** |
| --- |
| ≥ 65 years old |
| Hospitalized for rehabilitation after an acute medical condition |
| Expected duration of rehabilitation ≥ 2 weeks |
| Able to follow instructions to perform a Timed Up and Go test |
| Able to perform exercises |
| Willing to participate in 4 sessions of HIIT per week for 2 weeks |
| **Exclusion criteria** |
| Any acute condition involving tachycardia, hypotension, or fever |
| Abnormal cycle ergometer stress test result with   - - significant ECG modification   - decrease of more than 20 mmHg in blood pressure with exercise   - angina   - ST depression at a workload < 6 METs   - unable to reach an intensity of 15 out of 20 on the Borg scale of perceived exertion |
| Any unresolved acute medical or surgical condition of the following kinds:   - acute heart failure   - acute coronary syndrome in the last month   - active pericarditis/myocarditis/endocarditis   - thromboembolic disease with < 2 weeks of anticoagulation   - acute infection requiring intravenous treatment   - ongoing intravenous perfusion   - surgery in the last 2 months   - recent bone fracture |
| Any previous episode of primary cardiac arrest (i.e., cardiac arrest that did not occur in the presence of an acute myocardial infarction or during a cardiac procedure) |
| Severe heart failure (NYHA III and IV) |
| Severe stenotic or regurgitant valvular disease |
| Uncontrolled hypertension at rest (SBP ≥ 160 mmHg, DBP ≥ 100 mmHg) |
| Uncontrolled dysrhythmia |
| Non-sustained ventricular tachycardia with exercise |
| Implanted defibrillator |
| Obstructive cardiomyopathy |
| Resting HR > 100/min |
| Severe peripheral artery disease |
| Severe COPD (VEMS < 50%) |
| Exercise-induced asthma |
| Oxygen dependency |
| Musculoskeletal anomaly that may limit exercise participation |
| Delirium |
| Inability to follow instructions |
| Inability to give consent |


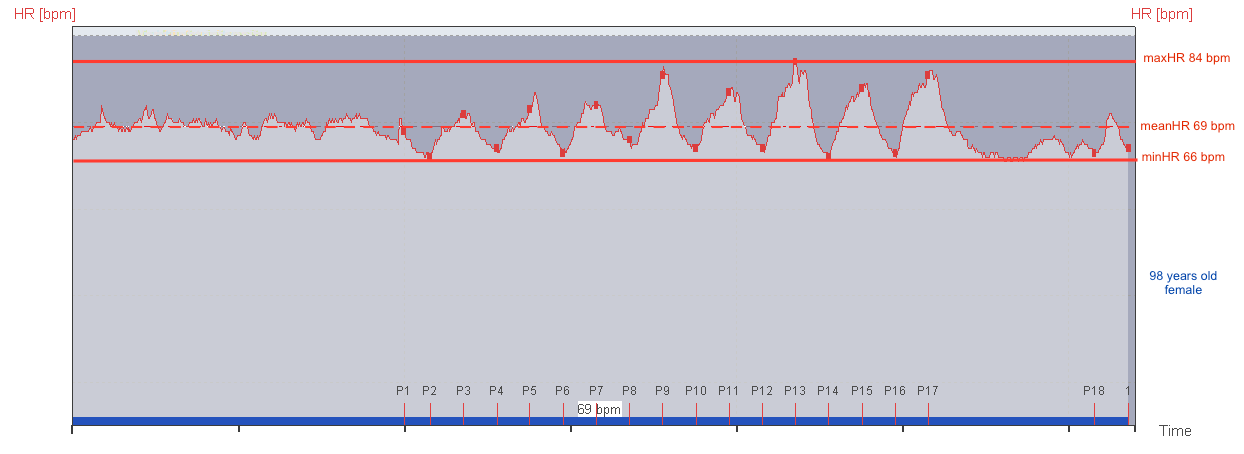


Additional Figure 1. HIIT session example 1

Figure 2. MICT session example 1


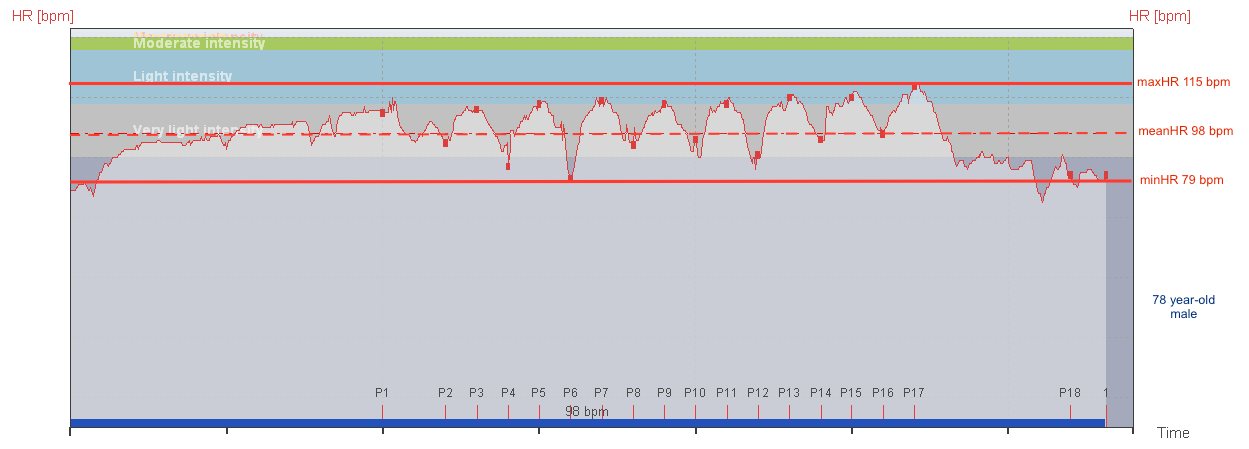


Additional Figure 2. HIIT session example 2

Additional Figure 3. MICT session example 1


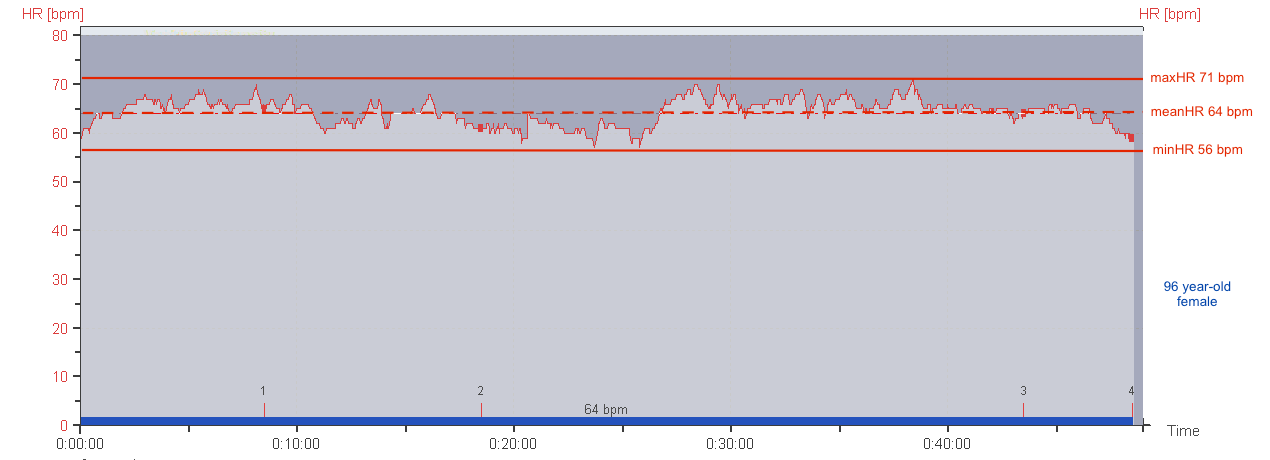


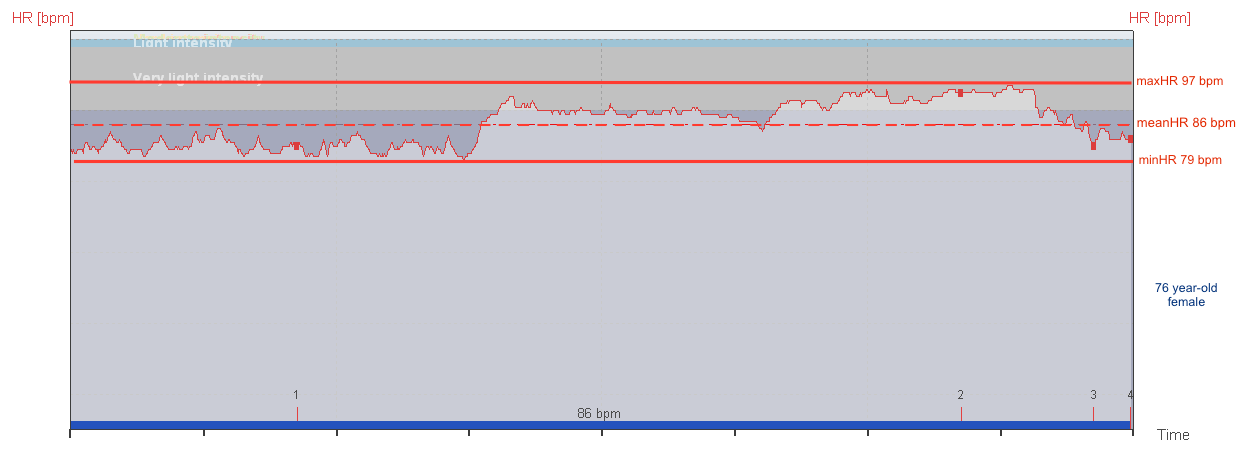


Additional Figure 4. MICT session example 2
